# Nintedanib (BIBF 1120) for IPF: a tomorrow therapy?

**DOI:** 10.1186/2049-6958-7-41

**Published:** 2012-11-12

**Authors:** Sabina A Antoniu

**Affiliations:** 1 Pulmonary Disease-Medicine II Department, University of Medicine and Pharmacy Grigore T Popa, Pulmonary Disease University Hospital, 30 Dr I CihacStr, Iasi, 700115, Romania

**Keywords:** Basic fibroblast growth factor, Idiopathic pulmonary fibrosis, Inhibitor, Platelet derived-growth factor, Tyrosine kinase, Vascular endothelial growth factor

## Abstract

Idiopathic pulmonary fibrosis is a rare, life threatening disease characterized by an anarchic fibrogenesis, limited survival and few therapeutic options. Its pathogenesis is complex and involves the interaction among various pathways driven by proinflammatory/profibrogenetic mediators such as platelet -derived growth factor, vascular endothelial growth factor or basic fibroblast growth factor. Given their prominent pathogenic roles in this disease such growth factor might be suitable therapeutic targets.In fact, the existing preclinical and clinical data demonstrated that their therapeutic inhibition results in a delayed progression of the pulmonary fibrosis and in the improvement of the disease outcome. BIBF 1120 is a potent triple blocker of the receptors of these growth factors which is currently evaluated as a potential therapy in the idiopathic pulmonary fibrosis. This review discusses the existing data supporting its potential use in this disease.

## Review

Idiopathic pulmonary fibrosis (IPF) is a rare life-threatening disease of the lungs characterized by a rapidly progressive fibrogenesis, limited survival and by limited therapeutic resources. The poor disease outcome is due not only to the fact that there are only few therapies available, but also to the rather modest antifibrotic effects of treatments such as corticosteroids, cyclophosphamide or azathioprine which are conventionally used as the first line approach in such patients [1]. More recently, pirfenidone, was approved as the first antifibrotic therapy for IPF based on demonstrated sustained clinical efficacy [2]. Other investigational drugs are currently assessed in the same setting and these include anticoagulant agents, anti growth factors agents or antioxidant molecules. Growth factor antagonists in particular are of promise due to their potent antifibrotic effects demonstrated in other similar settings [3,4].

BIBF 1120 is a triple tyrosin kinase inhibitor and a potent antagonist of growth factors such as platelet-derived growth factor, vascular endothelial growth factor and basic fibroblast growth factor, and it is currently evaluated in clinical trials as a potential IPF therapy.

This paper summarizes the rationale of its use in this disease based on existing evidence.

### Scientific rationale of tyrosin kinases inhibition in the IPF

Mediators such as platelet-derived growth factor (PDGF), vascular-endothelial growth factor (VEGF) and basic fibroblast growth factor (bFGF) are currently individually or collectively investigated as potential therapeutic targets for diseases such as cancers, pulmonary hypertension or idiopathic pulmonary fibrosis. VEGF, PDGF or FGF are now known mitogenic factors playing major pathogenic roles in tumour angiogenesis, extracellular matrix proliferation and fibrogenesis [5]. VEGF for example was found to be over expressed by tumour cells in response to tissue hypoxemia and was reported to increase proliferation and migration of the endothelial cells and to inhibit their apoptosis [6,7]. PDGF is another growth factor which was found to be involved in the pathogenesis of various proliferative disorders such as tumours, or pulmonary fibrosis in which it was found to stimulate proliferation of smooth muscle cells as well as fibrogenesis [6,8]. In a mouse model of silica-exposure-induced lung fibrosis, PDGF-BB was found to be over expressed via a TGF-β-mediated autocrine pathway [8].

bFGF was demonstrated to induce proliferation of smooth muscle cells, myofibroblasts, and fibroblasts and its expression was found to be up regulated in IPF [7].

Given the potent mitogenic effects of these growth factors, their inhibition is expected to reduce the angiogenesis and the fibrosis and in fact such effects are already used in cancer therapy and evaluated in various forms of cancer as well as in other diseases such as pulmonary fibrosis or IPF [9].

The inhibition of such factors can be achieved via different approaches, the most used and evaluated currently being the related receptors inhibition. VEGFR, PDGFR and bFGFR are tyrosine kinases which become activated upon ligand binding and stimulate the downstream signaling pathways [5].

Imatinibmesylate, another tyrosin kinase inhibitors which acts specifically on PDGF was initially evaluated for its potential antifibrotic effects in preclinical and clinical studies. In experimental studies imatinib was found to inhibit fibroblast proliferation, and collagen deposition *in vitro* and *in vivo*[10].

When evaluated in a clinical study in patients with IPF, imatinib given once daily at a dosage of 600 mg/day, for 96 weeks demonstrated no significant benefit over placebo in slowing disease progression or lung function impairment [11].

However, the multiple inhibition of such growth factors might be associated with a more potent antifibrotic effect: in a preclinical study performed in the mice model of bleomycin-induced pulmonary fibrosis, BIBF 1000 a triple kinase inhibitor for the PDGFR, VEGFR and bFGFR was found to attenuate bleomycin-induced lung fibrogenesis by reducing the expression of the pro-fibrogenetic factors and by decreasing the collagen lung content [12]. In a an ex vivo assay performed on human bronchial fibroblasts BIBF 1000 inhibited TGF-β-induced myofibroblast differentiation [12].

BIBF 1120 (nintedanib), an oxindole derivative is a triple kinase inhibitor with potent suppressing effects on VEGFR, PDGFR and bFGFR [9]. Nintedanib is currently in advanced clinical testing for various types of advanced solid cancers as a potential antiangiogenic therapy to be added to the cytotoxic agents to exert synergistic antitumour effects. The antiangiogenic effects of nintedanib were demonstrated in various preclinical studies such including inhibition of proliferation of HUVEC cell line or animal models of xenografttumour, as well as in clinical trials in subjects with advanced non-small cell lung cancer or gastrointestinal tumours [9].

### Clinical data with BIBF1120 in IPF

BIBF 1120 was assessed in many clinical studies performed in a wide range of solid malignancies including non-small cell lung cancer (NSCLC) as an antiangiogenic therapy and in few clinical studies as a potential antifibrotic therapy in IPF [9].

The TOMORROW (To Improve Pulmonary Fibrosis with BIBF-1120) study was a 12 month, randomized, placebo controlled, phase II study evaluating the efficacy and safety of four nintedanib doses (50 mg once daily, 50 mg twice daily, 100 mg twice daily, 150 mg twice daily) in patients with idiopathic pulmonary fibrosis [13].

Patients aged at least 40 years with a predefined IPF diagnosis of less than 5 years prior to the study screening were included in the study. Eligible patients had a FVC≥50% and a DL_CO_pred 30-79% and a PaO_2_ ≥55mmHg. Concomitant oral therapy with prednisone (or equivalents) of ≤15 mg if stable during the 8 weeks previous to the study enrollment [13].

The primary efficacy endpoint was represented by the FVC decline rate and the secondary endpoints included changes from baseline in FVC, DLco, SpO2, TLC, exercise capacity, SGRQ (St. George respiratory questionnaire) scores, the incidence of acute exacerbations, overall mortality, and that due to respiratory causes.

The highest BIBF 1120 dose was associated with the most significant therapeutic effect on the lung function decline compared to placebo, the drug reducing the annual rate of lung function decline by 68.4% compared to placebo group.

The highest dose of BIBF 1120 therapy was also associated with a lower percentage of patients exhibiting a significant reduction of the FVC (of >10% or of >200ml) compared to placebo (23.8% versus 44%, p=0.004). Unlike placebo BIBF 1120 preserved the total lung capacity (−0.24 liters vs. 0.12 liters, p<0.001).

Mean change from baseline in the SpO_2_ was −0.2% with BIBF 1120 and −1.3% with placebo (p = 0.02). The highest dose therapy was also associated with a lower percentage of significant desaturation (>4% reduction from baseline in resting SpO_2_) over the study period 3.6% respectively 11.0%, p = 0.03). BIBF 1120 didn’t exert a significant therapeutic benefit on the DL_CO_ and in the exercise capacity as compared to placebo.

Health Related Quality-of-Life (HRQoL) evaluated with SGRQ was found to be significantly improved with the BIBF highest dose as compared to placebo the difference being also clinically significant: mean change −0.66 points with the active treatment compared to 5.46 points with placebo p = 0.007. The domain analysis demonstrated that the significant therapeutic effect on HRQoL was due to the significant improvements in symptoms and activity domains (−3.14 points with BIBF 1120 compared to 6.45 points with placebo, p = 0.003 and 0.32 points with BIBF 1120 compared to 7.48 points, p = 0.004). A dose-dependent trend toward a reduction of the score of impact domain score reduction was also reported with the BIBF 1120. The proportion of patients with a clinically significant improvement in the HRQoL scores was higher in the treatment arms 100 mg and 150 mg (32.6% and 29.1%, respectively) versus placebo group (16.1%; p = 0.007 and p = 0.03, respectively). An in-depth analysis of the HRQoL and dyspnea change over baseline was also conducted. It was found a risk of dyspnea worsening versus baseline in a comparable number (%) of patients irrespective of the treatment allocation: 31(40.3%) in placebo arm, 31(41.3%) with BIBF-1120 50 mg/day 31(38.3%) BIBF-1120 100 mg/day, 33(40.2%) BIBF-1120 200 mg/day, 26(34.2%) BIBF-1120 300 mg/day. For the HRQoL however, a dose dependent improvement was demonstrated: 0.79 units with BIBF-1120 50 mg/day, 3.28 units with BIBF-1120 100 mg/day, 3.98 units with BIBF-1120 200 mg/day, 6.12 units with BIBF-1120 300 mg/day (p=0.0071 for the highest dose versus placebo). The overall HRQoL worsening in the placebo arm was also clinically significant (MCID 4 units of score). The most significant therapeutic benefit was detected in the SGRQ symptoms score: 9.6 units improvement with BIBF 1120 compared to placebo (p=0.0028). The likelihood of a clinically significant improvement was the highest with BIBF 1120 200 mg/day, (odds ratio 2.7, p=0.0069). Mean HRQoL change was found to correlate inversely with mean FVC change (r =0.0304, p=0.0081) [14]. BIBF 1120 highest dose was associated with a significant reduction of the incidence of acute exacerbations compared to placebo (2.4 versus 15.7 per 100 patient-years, p = 0.02), and a trend towards a dose-dependent increase in the effect size with the 100 mg dose was also noted. The 150 and the 100mg BIBF1120 doses were also associated with a trend toward a lower mortality rate compared to placebo (p = 0.04 for 100 mg and p = 0.06 for 150 mg). No significant differences in terms of mortality rates with the treatment groups compared to placebo were reported.

Overall, there were comparable incidences of adverse events among all groups with comparable rates of severe/life-threatening effects as well. The adverse events which most commonly led to treatment discontinuation were diarrhea, nausea, and vomiting and were reported in the group receiving 150 mg twice a day [13].

The TOMORROW trial is planned to be followed by an open-label continuation phase in which efficacy and safety of BIBF-1120 are planned to be further studied [15].

Two phase III studies aimed at evaluating the efficacy and safety of BIBF 1120 150 mg×2/day over 52 weeks in patients with IPF are also planned. These two studies have similar endpoints which include the annual decline rate in FVC, quality of life and time to first exacerbation, overall survival [16].

## Conclusions

IPF is a debilitating disease associated with a limited life span and characterized by an anarchic, rapidly progressing fibrogenesis. The pathogenesis of the IPF is complex and involves the interaction of multiple mediator-driven pathways having as common (up) regulator the TGF-β and including growth factors such as PDGF, bFGF or VEGF. The preclinical and clinical data demonstrated that the concomitant inhibition of such factors with compounds such as BIBF 1120 is able to slow the progression of lung fibrosis and to improve the disease outcome. However further clinical data are needed to better document the clinical efficacy and safety of BIBF 1120 and to better position this potential therapy in the IPF management.

## Competing interests

The author declares that she has no competing interests.
